# Characteristics of *Toxocara canis* induced lung inflammation in C57BL/6 mice

**DOI:** 10.3389/fimmu.2025.1597778

**Published:** 2025-08-21

**Authors:** Janina Lekki-Jóźwiak, Justyna Karabowicz, Magdalena Paschall, Karolina Gregorczyk-Zboroch, Małgorzata Sobczak-Filipiak, Piotr Bąska, Irma Schabussova, Ewa Długosz

**Affiliations:** ^1^ Institute of Veterinary Medicine, Department of Preclinical Sciences, Warsaw University of Life Sciences, Warsaw, Poland; ^2^ Institute of Veterinary Medicine, Department of Pathology and Veterinary Diagnostics, Warsaw University of Life Sciences, Warsaw, Poland; ^3^ Laboratory of Parasitology, Military Institute of Hygiene and Epidemiology, Warsaw, Poland; ^4^ Institute of Specific Prophylaxis and Tropical Medicine, Centre for Pathophysiology, Infectiology and Immunology, Medical University Vienna, Vienna, Austria

**Keywords:** toxocariasis, lung inflammation, eosinophils, cytokines, immune response

## Abstract

Toxocariasis, a neglected zoonotic disease caused by parasites of the *Toxocara* genus, represents a significant public health concern, with an estimated global seroprevalence of 19%. Despite the well-known respiratory symptoms associated with toxocariasis, the immune response in the lungs during toxocariasis is still poorly understood. This study analyzes both local lung and systemic immune response to *T. canis* infection and *T. canis* excretory-secretory antigens (TES) intranasal application in C57BL/6J mice. Lungs, blood, and spleens were collected at specific time points for histopathological analyses, flow cytometry, cytokine profiling, and gene expression studies. The systemic immune response was further assessed by cytokine measurements in splenocyte cultures and the detection of TES-specific antibodies. *T. canis* infection triggered severe pulmonary inflammation characterized by eosinophilia and mucus accumulation, with persistent inflammation lasting up to 28 days post-infection. Interestingly, this response was not solely driven by Th2-type interleukin production. Cytokine analysis of splenocyte cultures revealed elevated levels of IL-5 and IL-6, along with increased TES-specific IgE and IgG1 antibody concentrations. In contrast, TES application alone induced local eosinophil infiltration and upregulated genes associated with lung repair, though this response was less intense and shorter-lived compared to the infection. Our study is the first to present a comprehensive cytokine proteome analysis in mouse lungs during *T. canis* infection and stimulation by larval antigens, highlighting the key role of cytokines such as IL-5, IL-6, and IL-33. These findings provide new insights into the pathogenesis of toxocariasis and underscore the need for further research into potential therapeutic targets.

## 1 Introduction

Human toxocariasis is a globally significant parasitic disease that has been overlooked for decades, despite its widespread impact. A meta-analysis encompassing 71 countries across WHO regions reveals that nearly one-fifth of the world’s population - approximately 19%, or 1.41 billion individuals - are seropositive for *Toxocara* (1) The disease is particularly prevalent in tropical and subtropical regions but is also observed in Europe and North America, with average seroprevalence rates of 11% and 13%, respectively (2).

Toxocariasis is primarily transmitted via the fecal - oral route, typically occurring when humans accidentally ingest the infective eggs of *T. canis* or *T. cati*. While canines serve as the main hosts for *T. canis*, other species, including humans and mice, can act as paratenic hosts. Following ingestion in mice, *T. canis* larvae hatch in the stomach and penetrate the small intestine, starting visceral migration. By day one, they reach the liver, peaking on day two, then move to the lungs by day three. Between days 4 and 6, larvae spread to kidneys, heart, salivary glands, spleen, and muscles. Around day seven, they shift to the myotropic–neurotropic phase, mainly localizing in the brain and muscles. The number of larvae gradually decrease over time, although some can persist for up to a year. The migration pattern in mice is similar to humans, although the timing can be different (3). The symptoms of toxocariasis depend on the affected organs. The four recognized forms of toxocariasis include visceral larva migrans (VLM), ocular larva migrans (OLM), neurologic toxocariasis, and covert toxocariasis (4). One of the symptoms of VLM and covert toxocariasis is wheezing and coughing; however, our understanding of the pulmonary changes and the local immune response in the lungs associated with *T. canis* infection remains limited (5).

In addition to causing mechanical tissue damage and triggering an immune response through their migration, *T. canis* larvae produce excretory-secretory antigens (TES). These antigens modulate the host’s immune response, helping the larvae evade immune effector mechanisms and survive in the host’s tissues for years (6). TES is a complex mixture of various molecules, predominantly glycoproteins, which typically account for about 40% of its composition (7). Despite the significance of TES, their direct impact on the host organism, in the absence of active larvae, has not been described so far.

This study aimed to characterize both the local immune response in the lungs and the systemic immune response in mice during *T. canis* infection. Furthermore, it explored whether the administration of TES antigens alone can trigger an inflammatory response. In conclusion, this study aims to clarify the function of TES in the context of *T. canis* infection.

## 2 Materials and methods

### 2.1 Parasites and TES collection

Adult *T. canis* worms were collected from the feces of dewormed dog treated in veterinary clinics in Warsaw. Eggs were obtained from six dissected female worms and incubated in 1% paraformaldehyde at room temperature. The embryonation process was monitored microscopically.

Fully embryonated eggs were hatched *in vitro* using the following protocol. Eggs were washed three times with 5 mL of sterile 0.85% NaCl followed by centrifugation (500 × g, 5 min.). Then egg pellet was resuspended in 5 mL of 10% solution of commercial bleach dissolved in 0.85% NaCl and incubated for 15 minutes on a roller shaker at room temperature. After centrifugation (500 × g, 5 min.) the residues of bleach were removed by two washes in 5 mL of 0.85% NaCl followed by two washes in 5 mL of Minimal Essential Medium (Sigma-Aldrich) supplemented with penicillin (100 U/ml), streptomycin (100 μg/ml), and amphotericin B (2.5 μg/ml) (MEM/A). After washing, eggs were suspended in 0,5 mL of MEM/A, placed on a 40 µm nylon mesh strainer in a 6-well tissue culture plate containing 5 mL of MEM/A, and incubated at 37°C, and 5% CO_2_. After three days hatched larvae were collected from the bottom of the plate, washed with 1 mL of MEM/A and transferred to a new 24-well plate at approximately 1,000 larvae per well. In order to collect TES, the plate was incubated for several weeks (37°C, 5% CO_2_). The culture medium was replaced every 3 days. The spent medium containing TES was collected and stored (-20 °C) until used. Upon multiple media changes, the frozen batches were pooled (totaling around 50 mL), concentrated, and dialyzed against sterile phosphate-buffered saline (PBS) using Amicon Ultra Centrifugal Units with 3 kDa cut-off (Merck Millipore). TES solution was filtered through a 0.22 μm filter, and antigen concentration was determined using BCA Protein Assay (Thermo Scientific).

### 2.2 Mice

Female C57BL/6J mice were purchased from Charles River (Sulzfeld, Germany). Mice aged 6 to 8 weeks were maintained under standard conditions with a 12-hour light-dark cycle and had access to water and food ad libitum. All procedures performed in this study involving animals were accepted and conducted in accordance with the guidelines of the Second Local Ethical Committee at Warsaw University of Life Sciences (Approval no. WAW2/094/2022).

### 2.3 Experimental design

**Figure 1:**
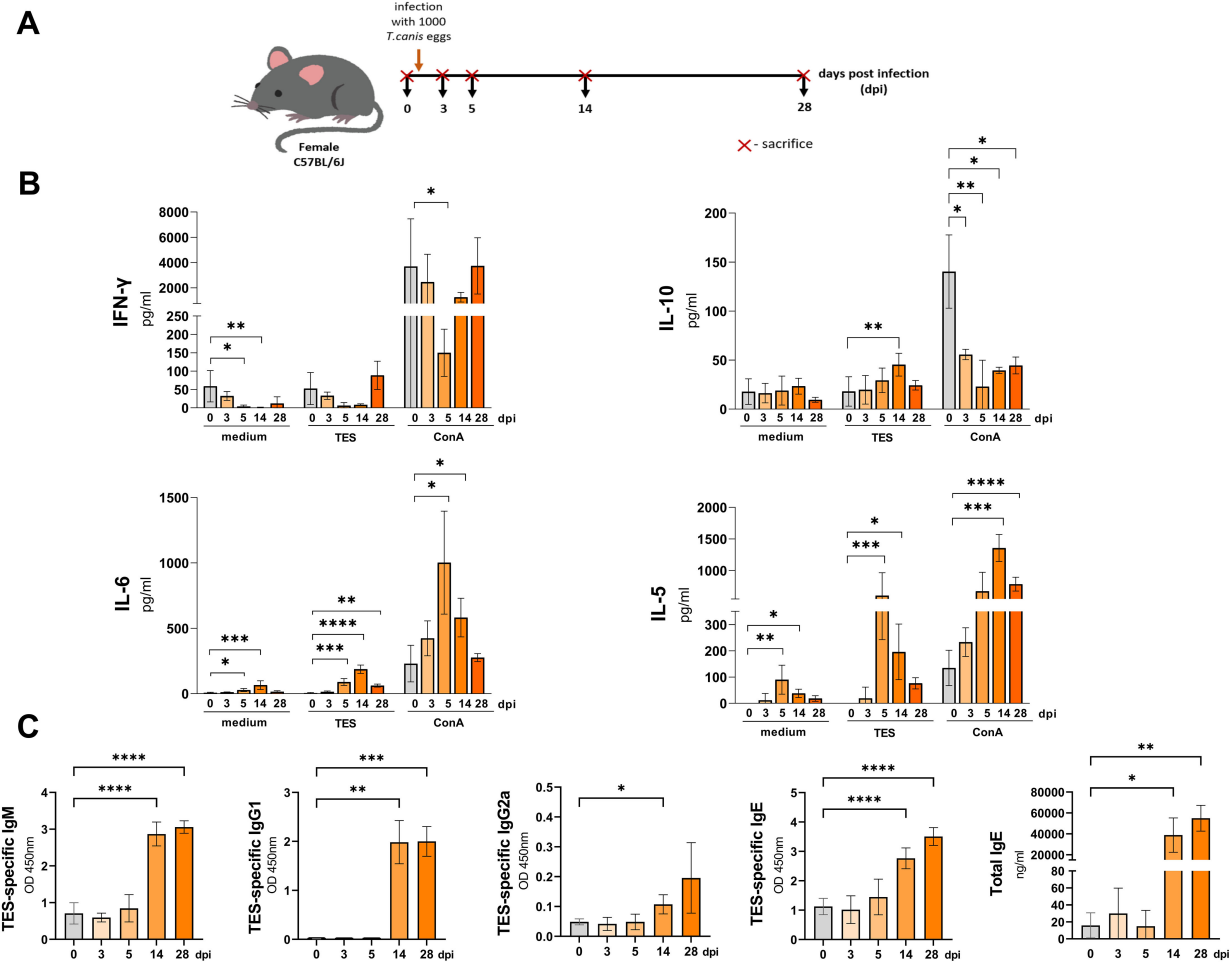
Cytokine and antibody response in mice infected with *T. canis*. **(A)** Schematic timeline representing the infection in C57BL/6J mice. **(B)** Splenocyte cytokine response in unstimulated (medium) and TES (1ug/ml) or ConA (5 ug/ml) stimulated cultures. **(C)** TES specific and total IgE antibody levels in mouse sera. Data is presented as mean ± SD from 5 mice in each group. Statistical differences compared to uninfected mice (0 dpi) are shown: *p < 0.05; **p < 0.01, ***p < 0.001, ****p < 0.0001. dpi – days post infection.

Two different experiments were conducted. In the first experiment, eight-week-old female C57BL/6J mice (n = 20) were orally infected with 1,000 infective eggs of *T. canis* suspended in 20 µL of 0.9% NaCl solution. Non-infected mice (n = 5) served as controls. Five infected mice were euthanized at 0 (control group), 3, 5, 14, and 28 days post-infection (dpi) (**Figure 1A**). 

In the second experiment, eight-week-old female C57BL/6J mice (n = 18) were treated intranasally with 2 μg of TES in 20 μl of PBS (10 µL per nostril), while control mice (n = 5) received intranasal treatment with the same volume of PBS. Nasal administration was applied once per day for 3 days. Six TES-treated mice were euthanized at each of the following time points: 3, 14, and 28 days post-first intranasal administration (dpfi). The control mice were euthanized at 3 dpfi.

### 2.4 Sample collection

Blood was collected and left to coagulate. After centrifugation (400 × g, 10 min), the sera were collected and stored at -20°C until used. Spleens were removed, submerged in sterile PBS buffer, and placed on ice until splenocyte isolation. Lungs were removed, the left lung was used for cell isolation, and the right lung was cut into three pieces. One was placed in paraformaldehyde for pathological study. The second was snap-frozen in liquid nitrogen for RNA isolation. The third lung fragment was homogenized in a sterile PBS-containing protease inhibitor cocktail (Sigma-Aldrich). After homogenization, Triton X-100 was added to a final concentration of 1%, and the samples were frozen at ≤ -70°C until Proteome Profiler Arrays (R&D Systems) were performed.

### 2.5 Cell isolation and culture

The left lung lobe was cut into small pieces and placed in 3 ml of digestion buffer containing Liberase TL (Roche) (10 µg/ml), DNaseI (Roche) (0.5 mg/ml), and gentamycin (Gibco) (100 µg/ml) diluted in RPMI 1640 medium (Biowest). The samples were incubated for 30 minutes at 37°C with continuous agitation. Afterward, the digested lungs were placed on a 70µM nylon mesh strainer primed with RPMI 1640 containing 100 µg/ml gentamycin and mashed through with the rubber end of a syringe plunger and rinsed thoroughly with RPMI 1640/gentamycin. Cells were then centrifuged at 300 × g for 5 minutes. The supernatant was discarded, and cells were suspended in 1 ml of Red Blood Lysis Buffer (Roche). After 90 seconds, 5 ml of PBS was added, and cells were centrifuged (300 × g, 5 min). The cell pellet was then suspended in 3 ml of PBS containing 2% fetal bovine serum (FBS) (Biowest). Cells were mixed with trypan blue and counted using a hemocytometer. An appropriate number of cells was used for cytometric analysis.

For splenocyte isolation, spleens were mashed through a 70µM nylon mesh strainer primed with RPMI 1640 supplemented with 100 µg/ml gentamycin and centrifuged (300 × g, 5 min). The supernatant was discarded, and erythrocytes were depleted as described above. The cell pellet was then suspended in 3 ml of complete RPMI 1640 culture medium containing 10% FBS, 100 µg/ml gentamycin, and 0.5 mM β-mercaptoethanol, followed by determining cell number and viability. Splenocyte suspension (5 × 10^6^/ml) was then transferred to a 96-well culture plate (200 µl per well) and stimulated with concanavalin A (5 µg/ml) or TES (1 µg/ml). Unstimulated cells served as controls. Splenocytes were incubated at 37°C with 5% CO_2_ for 72 h. Then, the culture medium was removed and stored at -20°C.

### 2.6 Pathological study

Samples were embedded in paraffin (Paraplast, Sigma-Aldrich), and subsequently cut on a rotatory microtome. Preparations of 4 µm thickness were automatically stained with hematoxylin and eosin (HE) or Periodic Acid–Schiff (PAS) using a Varistain Gemini autostainer (Thermo Scientific, UK).

The microscopic analysis of HE and PAS slides was carried out with a BX43 light microscope equipped with an SC30 digital camera (Olympus Optical, Japan). Pictures were analyzed and recorded by computer software (CellSens Entry 2011, Olympus Lifescience).

### 2.7 Flow cytometry

Multiparametric flow cytometric analyses were performed in isolated lung cell suspensions. Two panels were used. The myeloid cells were identified using the following antibodies: FITC anti-mouse Ly-6G/Ly-6C (Gr-1) (1:200), Alexa Fluor 700 anti-mouse CD11c (1:200), PE/Cyanine 7 anti-mouse I-A/I-E (1:300), PE/Dazzle 594 anti-mouse DC170 (Siglec F) (1:200), APC/Cyanine 7 anti-mouse Ly-6C (1:200). The lymphoid cells were stained using: FITC anti-mouse TCRβ chain (1:250), PE/Dazzle 594 anti-mouse CD49b (pan-NK cells) (1:200), APC/Cyanine 7 anti-mouse CD4 (1:200), Pacific Blue anti-mouse CD8a (1:200). Moreover, TruStain FcX (anti-mouse CD16/32) (1:50) and Zombie Aqua Fixable viability kit (1:500) were used in both panels. All reagents were from BioLegend.

A number of 1 × 10^6^ of isolated lung cells was used for staining using each antibody panel. First, cells were washed three times by suspending cell pellets in PBS buffer and centrifuging (310 × g, 5 min). Then cells were incubated in TruStain FcX (CD16/32) antibody diluted 1:50 in PBS for 15 minutes, washed with PBS and incubated in ZombieAqua Fixable viability dye diluted 1:500 in PBS for another 15 minutes. Then, the cells were washed with PBS containing 2% FBS. After washing, cells were stained for 15 minutes with antibodies, separately for the myeloid panel and for the lymphoid panel. Then, the cells were washed and resuspended in PBS supplemented with 2% FBS. Data was acquired on a BD LSR II flow cytometer using BD FACSDiva software (BD Biosciences). Compensation and data analyses were performed using FlowJo software (TreeStar, Ashland, OR). Cell populations were identified using sequential gating strategy, and the percentage of cells in the live/singlets gate was established.

### 2.8 Proteome profiler analysis

The lung proteome was analyzed using the Proteome Profiler™ Mouse XL Cytokine Array (R&D Systems, USA). First, the total protein concentration was measured in each tissue lysate using Pierce BCA Protein Assay Kit (Thermo Scientific). Equal amounts of protein from each lung lysate sample were collected and pooled by group, with a total of 150 µg of protein per group. A separate membrane was used for each group. Due to a limited number of proteome arrays one group (14 dpfi) had to be excluded. The results were measured using ImageJ, and the Integrated Density value was used for analysis.

### 2.9 Cytokine measurements

Levels of IL-4, IL-5, IL-6, IL-10, IL-13, IL-17, IL-33, IFN-γ, TGF-β, and TNF-α were measured in splenocyte culture media and lung tissue lysates (IL-13 was measured only in lung tissue lysates) using Mouse DuoSet ELISA kits (R&D Systems) according to manufacturer’s instructions with slight changes. The reaction was performed in a working volume of 50 µl instead of 100 µl, and the stage of standard and sample incubation was prolonged to 16 h at 4 °C. The absorbance was measured with a Synergy H1 ELISA microplate reader (BioTek Instruments, USA) at 450 nm, with corrections made at 570 nm. A four-parameter logistic (4-PL) curve was generated, and cytokine concentrations were calculated using BioTek Gen5 software version 3.12 (Agilent Technologies, USA).

### 2.10 Antibody ELISA

TES-specific IgG1, IgG2, IgE, and IgM immunoglobulins were measured in sera of experimental mice using ELISA. Each well of the 96-well plate was coated with 100 µl TES (0.1 µg/ml in PBS), and incubated overnight at 4°C. After incubation, the plates were washed three times with PBS containing 0.05% Tween-20 (PBS-T) and subsequently blocked with 300 µl of 5% non-fat milk in PBS at 37°C for 1 hour. Following the blocking step, the plates were washed three times with PBS-T and diluted serum samples from each mouse were added and incubated at 37°C for 1 hour. For IgG1 and IgG2 measurements, serum samples were diluted in PBS at 1:100, for IgM at 1:500, and for IgE at 1:250. After incubation, the plates were washed three more times with PBS-T. Next, HRP-conjugated goat anti-mouse IgM, IgE, IgG1, and IgG2a (Bio-Rad) were added as secondary antibodies at a 1:4000 dilution in PBS, followed by incubation at 37°C for 2 hours. The plates were washed, and 100 µl of TMB substrate (Thermo Fisher Scientific) was added to each well. The plates were incubated in the dark for 20 minutes following reaction termination using 50 µl 2M H_2_SO_4_. Finally, the absorbance was measured at 450 nm using the Synergy H1 ELISA microplate reader (BioTek Instruments, USA).

The level of specific anti-TES IgE antibodies was also measured in lung tissue lysates. The procedure was similar to the one described above, except that lysates were diluted in PBS to contain 50 µg/well or 20 µg/well in case of 14 and 28 dpi groups and used instead of serum samples. The secondary antibody was diluted 1:10,000.

Total IgE in serum was analyzed using Mouse IgE ELISA Antibody Pair Kit (StemCell Technologies) according to the manufacturer’s instructions. All samples were diluted 1:100, except for 14 and 28 dpi, which were diluted 1:100,000. The dilutions were prepared in PBS-T and 0.1% bovine serum albumin. The intensity of the enzyme reaction was measured at 450 nm using the Synergy H1 ELISA microplate reader (BioTek Instruments). Total IgE concentration was calculated using a standard curve generated using standard mouse IgE provided by the manufacturer.

Total IgE levels were also measured in lung tissue lysates using the same antibody kit protocol. Tissue lysates were used instead of sera, and diluted in PBS to contain 50 µg of lung protein per well, except for the 14 and 28 dpi samples, where 5 µg of protein per well was used. Total IgE concentration, which was calculated using the standard curve, was then recalculated per 1 mg of lung tissue.

### 2.11 Reverse transcription quantitative PCR

Total RNA was isolated from the samples using the Total RNA Mini Kit (A&A Biotechnology), followed by measurement of RNA concentration using a spectrophotometer. Residues of genomic DNA were removed using DNase I treatment (Thermo Fisher Scientific). The reaction mixture was prepared as follows: 1µg RNA solution, 1.1 µl of 10 × DNAse I buffer, 1.1 µl of DNAse I (1U/µl), water (up to 12.1 µl). The mixture was incubated at 37°C for 40 min, followed by adding 50 mM EDTA (1.1 µl) and incubation at 65°C (10 min). The negative control containing all the above mentioned solutions, except RNA, was prepared parallelly. Eleven µl of each reaction containing RNA from the investigated samples were subjected to reverse transcription as follows: 1 µl of poly-T primer (100 µM) was added to RNA solution followed by incubation at 65°C for 5 min and immediately chilled on ice. Eight µl of the precooled mix containing 4 µl of 5 × buffer, 2 µl of dNTPs mix (10 mM), and 1 µl of RevertAid Reverse Transcriptase (200 U/μL) was added to each sample followed by incubation at 42°C (60 min). The reaction was terminated by incubation at 70°C for 5 minutes. Eleven µl of negative control reaction was subjected to a similar protocol, but instead of dNTPs and poly-T primer, water was added. All the investigated samples and the prepared negative control were diluted 40X used as matrices for QT - PCR, which was performed using Maxima SYBR Green qPCR Master Mix (2X) (ThermoFisher) in Agilent Stratagene Mx3005P Quantitative PCR System. The mixture contained 6 µl of Maxima SYBR Green qPCR Master Mix (2X), 0.72 µl of primer mix (5 µM forward and 5 µM reverse primers) (**Table 1**), 0.6 µl of ROX reference dye (200 nM) (ThermoFisher), 2.68 µl of water, and 2 µl of template. The thermal profile of the reaction was as follows: 95°C - 10 min, 45 cycle rounds (95°C - 15 s, 60°C - 1 min) followed by the melting curve acquisition. Gene expression was quantified using the 2^−ΔΔCT^ method (8), with *Hprt* as the reference gene for expression normalization.

**Table 1:** Description of primers used in RT-qPCR.

Gene	Forward primer	Reverse primer
*Amphiregulin*	ACTTTGGTGAACGGTGTGGA	CACTGTGATAACGATGCCGA
*Arginase-1*	TCCAGAAGAATGGAAGAGTCAG	CAGATATGCAGGGAGTCACC
*Chil3l1*	GGAGTGGAATGACGAGTCGA	CAGTGTTGGAGGCAATCTCG
*Chil3l3*	TTTCTGAATGAAGGAGCCACTGA	GAGCCACTGAGCCTTCAACT
*Fizz1*	GTGAATACTGATGAGACCATAGAGAT	GCAGTGGTCCAGTCAACGAG
*Hprt*	AGACTGAAGAGCTACTGTAATGATCA	GGTCCTTTTCACCAGCAAGCT
*Osteopontin*	ACACACAGACTTGAGCATTCC	TCCTTGTGGCTGTGAAACTTG
*Reg3g*	TTCTCAGGTGCAAGGTGAAGTT	ACTGAGCACAGACACAAGATGT
*Tgfb1*	ACCGCAACAACGCCATCTAT	GTAACGCCAGGAATTGTTGC

### 2.12 Statistical analysis

Group comparisons were conducted as follows: the normality of the data distribution was assessed using the Shapiro-Wilk test, followed by an evaluation of variance equality using the Brown-Forsythe test, respectively. For groups with normally distributed data and homogeneous variances, one-way ANOVA was performed, followed by Dunnett’s *post-hoc* multiple comparisons test. Groups with normally distributed data but unequal variances were analyzed using Welch’s ANOVA, followed by Dunnett’s T3 *post-hoc* multiple comparisons test. Groups without normally distributed data were analyzed using the Kruskal-Wallis test, followed by Dunn’s *post-hoc* multiple comparisons test. All analyses were conducted using GraphPad Prism 9.5.1, and results were considered statistically significant at p < 0.05.

## 3 Results

### 3.1 *T. canis* infection induces systemic IL-5 and IL-6 production

The schematic diagram of T. canis infection in mice is shown in **Figure 1A**. To evaluate the splenocyte cytokine response, we measured IL-4, IL-5, IL-6, IL-10, IL-17, IL-33, IFN-γ, TGF-β and TNF-α levels using ELISA. IL-4, IL-17, IL-33, TNF-α were below the detection limit. No significant differences in TGF-β concentrations were observed between infected and control mice (data not shown).

Splenocytes isolated from experimental mice spontaneously released small amounts of IL-10 throughout the experiment, with no significant variation across time points (**Figure 1B**). IFN-γ secretion was down regulated in cultures from infected mice, particularly at 5 and 14 dpi, where reductions were statistically significant. In contrast, spontaneous IL-5 and IL-6 release was significantly higher in these cultures compared to control mice.

Restimulation of splenocytes isolated from infected mice with TES led to a marked upregulation of IL-10 at 14 dpi, IL-6 at 5, 14, and 28 dpi, and IL-5 at 5, and 14 dpi (**Figure 1B**). Additionally, IL-10 secretion by splenocytes from all infection time points was significantly inhibited after ConA stimulation compared to controls. IFN-γ secretion was also lower but reached statistical significance only at 5 dpi. Conversely, ConA-treated splenocyte cultures exhibited significantly higher IL-6 production at 5, and 14 dpi, and increased IL-5 secretion at 14, and 28 dpi.

Additionally, to evaluate the systemic immune response to *T. canis* infection, we analyzed changes in serum TES-specific antibodies and quantified total IgE levels. TES-specific IgE and IgM levels significantly increased from 14 dpi, and remained elevated through 28 dpi (**Figure 1C**). IgG1 also rose significantly at 14 dpi, peaking at 28 dpi, whereas IgG2a exhibited a mild increase at 14 dpi but no significant up-regulation at 28 dpi. Similarly, total IgE increased at 14 dpi, reaching its highest levels at 28 dpi.

### 3.2 *T. canis* infection leads to severe lung inflammation

**Figure 2:**
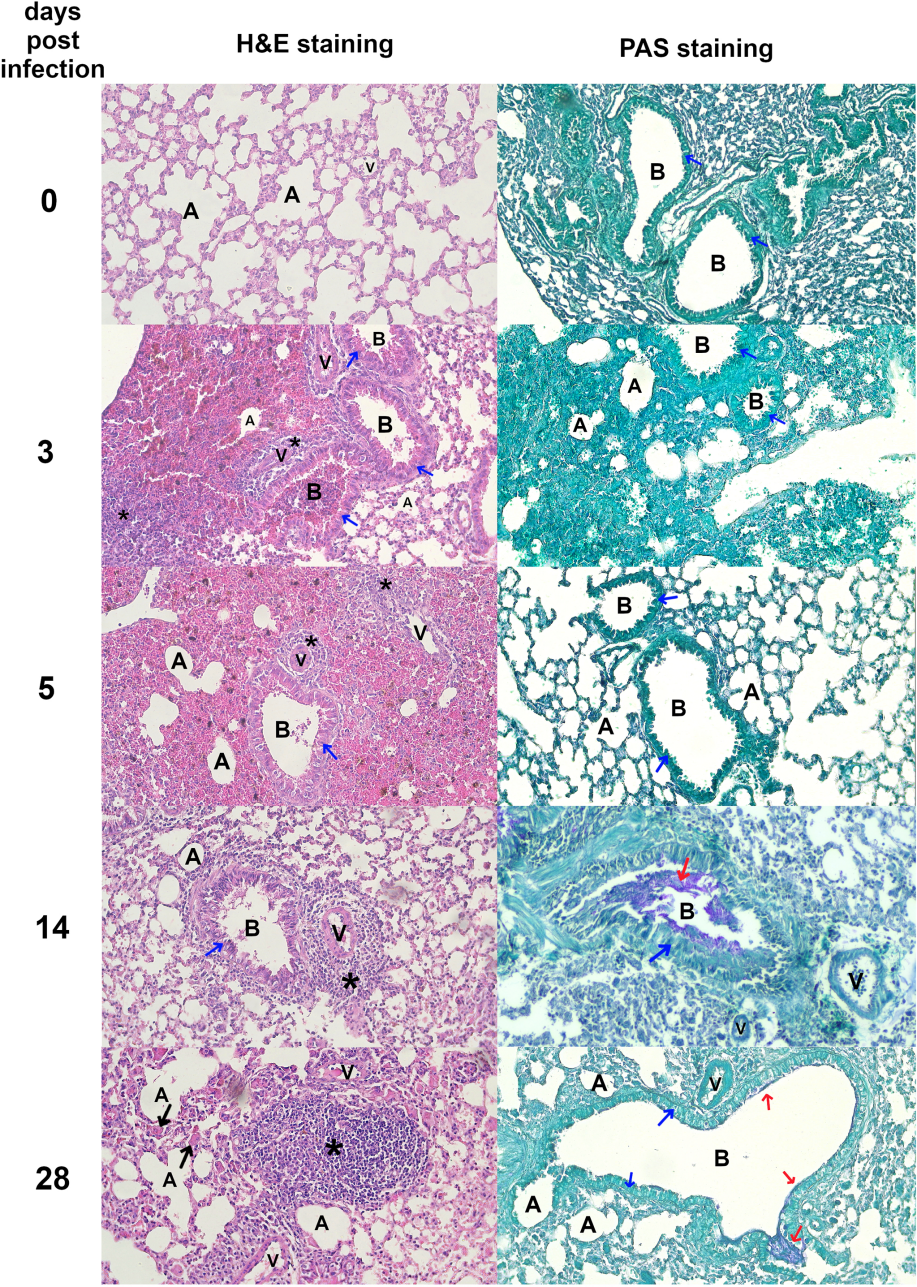
Histopathological changes in *Toxocara canis* infected mouse lungs Representative images of lung sections stained with H&E and PAS are shown (magnification ×200). Figure legend: A, alveolar lumen; B, bronchiole; V, blood vessel; * – cellular infiltrate; blue arrow – bronchial epithelial cell; red arrow – mucus; black arrow – giant cell. Pictures illustrating data from all experimental animals are available at https://doi.org/10.18150/83F56M.

**Figure 3:**
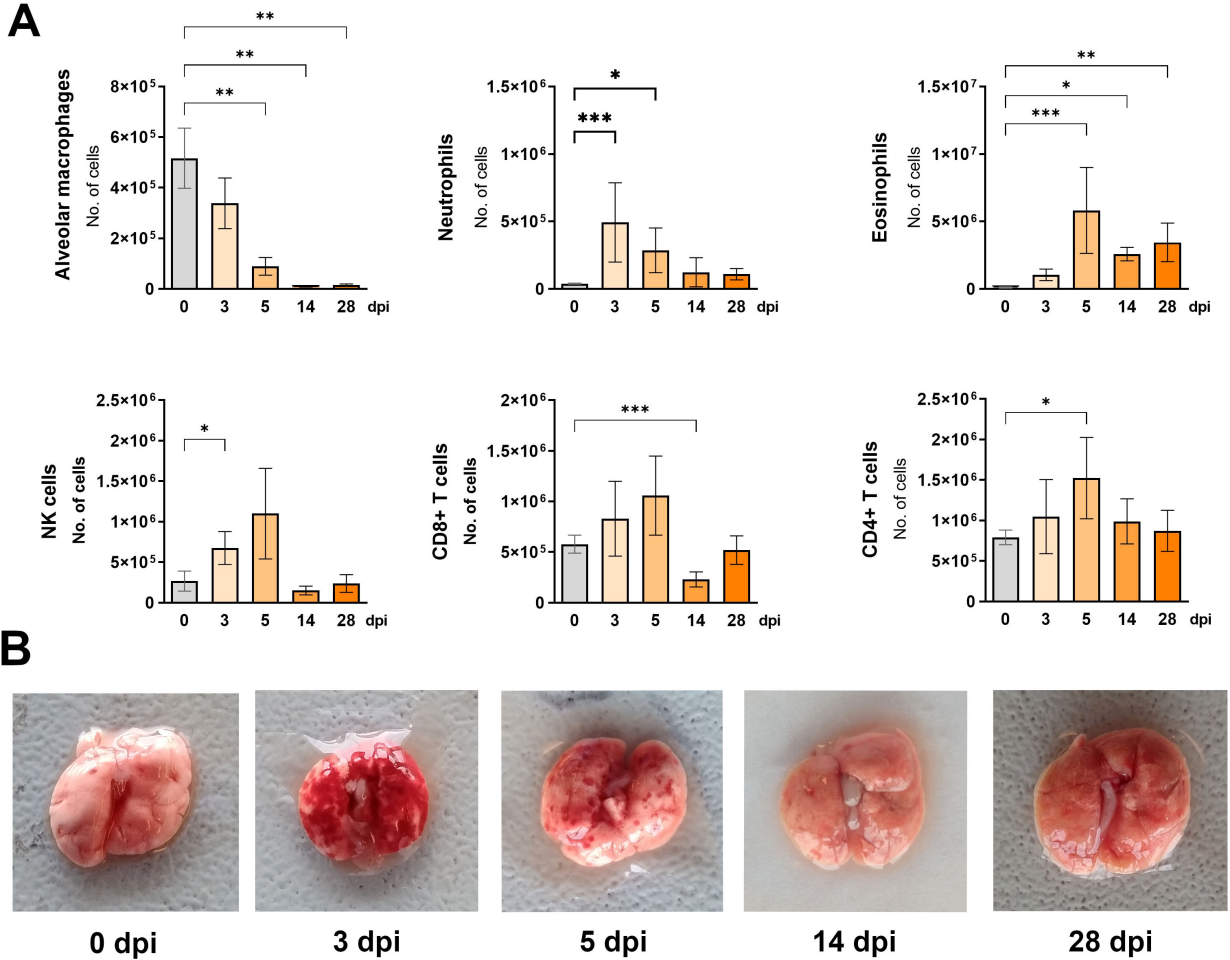
Changes in lung cell populations during *Toxocara canis* infection in mice. **(A)** Absolute numbers of different lung cell populations determined using flow cytometry: neutrophils (Ly6G+CD11b+SiglecF-); alveolar macrophages (CD11c+SiglecF+); eosinophils (Ly6G+CD11b-SiglecF+); NK cells (CD49+); T helper lymphocytes (TCRαβ+CD4+); T cytotoxic lymphocytes (TCRαβ+CD8+). Data is presented as mean ± SD from 5 mice in each group. Statistical differences compared to uninfected mice (0 dpi) are shown: * p < 0.05; **p < 0.01, ***p < 0.001. dpi, days post infection. **(B)** Representative macroscopic pictures of mouse lungs at different infection timepoints.

Morphological and histopathological analysis of the lungs in *T. canis*-infected mice revealed a progressive response to infection over time. **Figures 2** and **3B** show histopathological analysis and representative pictures of lungs from each group. Pictures illustrating data from all experimental animals are available at RepOD Repository for Open Data (https://doi.org/10.18150/83F56M). In control animals (0 dpi), the lungs appeared normal, with only a few macrophages, neutrophils, mast cells, and scattered lymphocytes around blood vessels, bronchioles, and in the alveolar lumen. At 3 dpi, the lungs exhibited visible inflammation, congestion, and mixed inflammatory cell infiltration (**Figure 2**). Hemorrhages around blood vessels and bronchioles, vacuoles in bronchiolar epithelial cells, and erythrocytes in the bronchi indicate an acute inflammatory response. At 5 dpi, the redness had decreased, but congestion and focal hemorrhages persisted. Inflammatory cells, particularly eosinophils and mast cells, infiltrated around blood vessels and bronchioles, indicating an evolving immune response. At 14 dpi, signs of healing were evident, with reduced redness but ongoing congestion and inflammation. Neutrophils, eosinophils, and macrophages predominated, and giant cells appeared, indicating chronic inflammation and tissue damage. By 28 dpi, the lungs showed signs of recovery with reduced redness, although significant macrophage infiltration persisted. Giant cells remained, and lymphoid nodules began to form, reflecting ongoing resolution and a chronic immune response. PAS staining revealed mucus accumulation in the bronchial lumen starting from 14 dpi. Overall, the histopathological analysis showed a shift from an early acute inflammatory response to chronic inflammation, with thexrecruitment of eosinophils, macrophages, and the formation of giant cells and lymphoid structures throughout the infection (**Figure 2**).

The presence and number of immune cells in the lungs of infected mice were also assessed by flow cytometry (**Figure 3A**). At 3 dpi, a significant number of neutrophils and NK cells were observed. Two days later, eosinophils and CD4+ T cells increased while alveolar macrophage numbers began to decline. Four weeks post-infection, neutrophil, NK cell, and lymphocyte numbers returned to control levels, whereas alveolar macrophages remained significantly reduced, and eosinophils were still increased.

**Figure 4:**
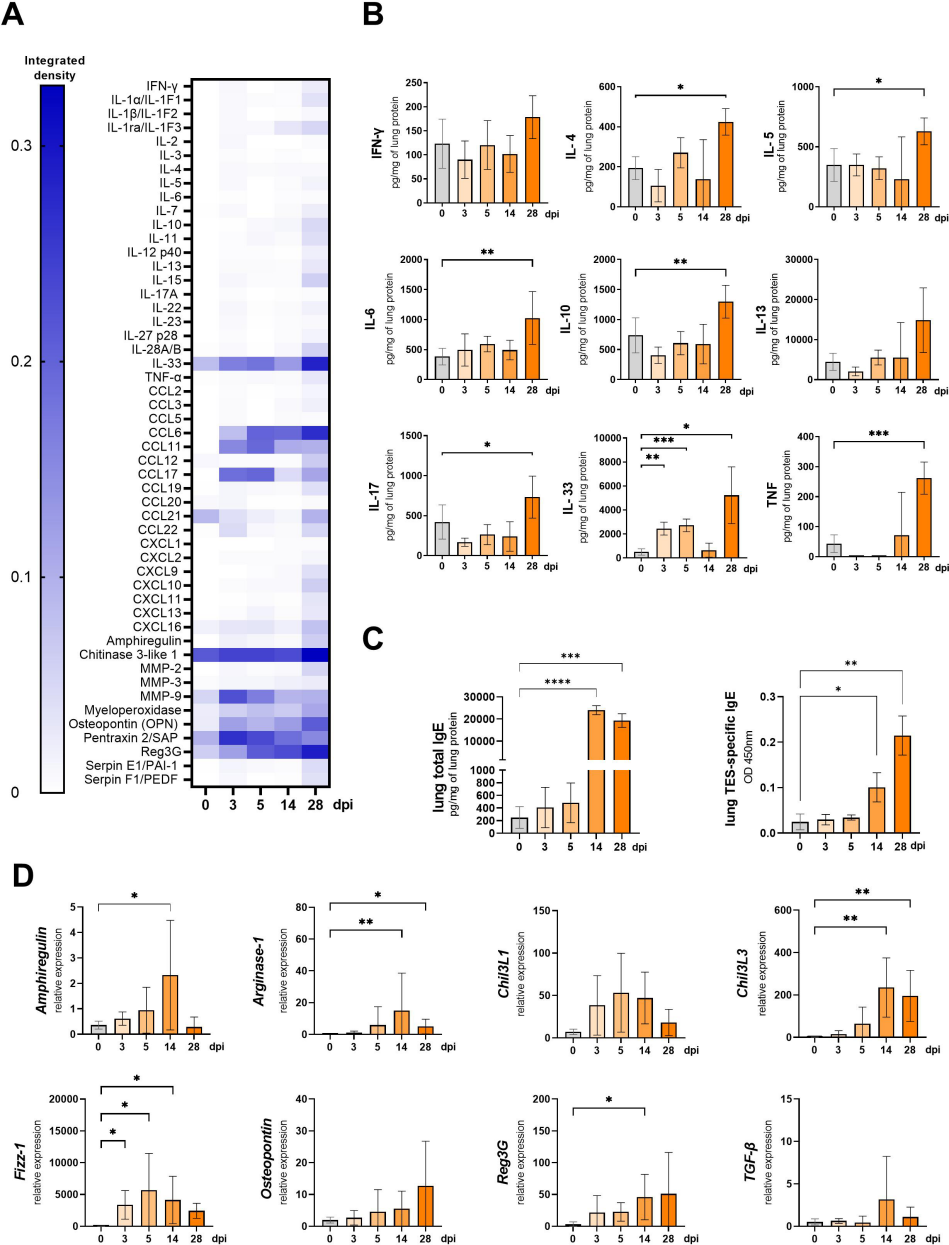
Cytokine expression and antibody changes in lungs of *Toxocara canis* infected mice. **(A)** Heat map representing cytokine expression in lungs determined using Proteome Profiler Mouse Cytokine XL assay. **(B)** Cytokine concentrations in lung tissue lysates measured using ELISA. **(C)** Total IgE and TES-specific IgE measured in lung tissue homogenates. **(D)** qPCR analysis of relative expression of selected genes involved in lung inflammation/tissue remodeling. Results are represented as relative expression to the *Hprt* housekeeping gene. Data **(A, C, D)** is presented as mean ± SD from 5 mice in each group. Statistical differences compared to uninfected mice (0 dpi) are shown: *p < 0.05; **p < 0.01, ***p < 0.001, ****p < 0.0001. The heat map **(B)** shows the mean of the integrated pixel density of two duplicate spots representing each analyte. dpi, days post infection.

To further characterize the local immune response in the lungs of infected mice, a Proteome profiler was used to screen for changes in cytokine expression. **Figure 4A** shows a heat map representing the most prominent changes in cytokines expression associated with lung inflammation and remodeling. The complete dataset is available at RepOD Repository for Open Data https://doi.org/10.18150/83F56M). The findings were validated through ELISA measurements of interleukins in lung lysates (**Figure 4B**) and qPCR analysis of fibrosis-related cytokines (**Figure 4D**).

*T. canis* migration through mouse lungs led to an increase in chemokines, particularly CCL17, CCL6, and CCL11, while CCL21 levels decreased at 3 and 5 dpi (**Figure 4A**). Aside from IL-33, no significant upregulation in other cytokines was detected at early infection stages, as confirmed by the Proteome profiler and ELISA. However, by 28 dpi, levels of IL-4, IL-5, IL-6, IL-10, IL-17, and TNF-α were significantly elevated (**Figures 4A, B**).

The Proteome profiler analysis revealed that throughout the infection, pentraxin, osteopontin, myeloperoxidase, and chitinase 3-like 1 (Chil3L1) levels remained elevated compared to 0 dpi, with an early increase in matrix metalloproteinase-9 (MMP-9) expression observed at 3, and 5 dpi, and a continuous rise in regenerating islet-derived protein 3 gamma (Reg3G). By 28 dpi, nearly all analyzed proteins were upregulated (**Figure 4A**). qPCR analysis (**Figure 4D**) confirmed the increased expression of *Amphiregulin*, *Osteopontin*, *Chil3L1*, and *Reg3G*, though only *Reg3G* upregulation at 14 dpi was statistically significant.

We have also analyzed the expression of additional genes involved in M2 macrophage polarisation. The expression of *Fizz-1* was up-regulated starting from 3 dpi and remained significantly elevated through the experiment, except at 28 dpi, where the change was insignificant. *Arginase-1* and *Chil3L3* were significantly upregulated at 14 and 28 dpi. Surprisingly, no changes were observed in TGF-β expression (**Figures 4B, D**).

Furthermore, IgE antibody levels in lung tissue lysates were measured by ELISA (**Figure 4C**). Total IgE levels significantly increased at 14 dpi and were further upregulated at 28 dpi, similarly as the TES-specific IgE levels.

### 3.3 Intranasal TES stimulation activates the systemic immune response

**Figure 5:**
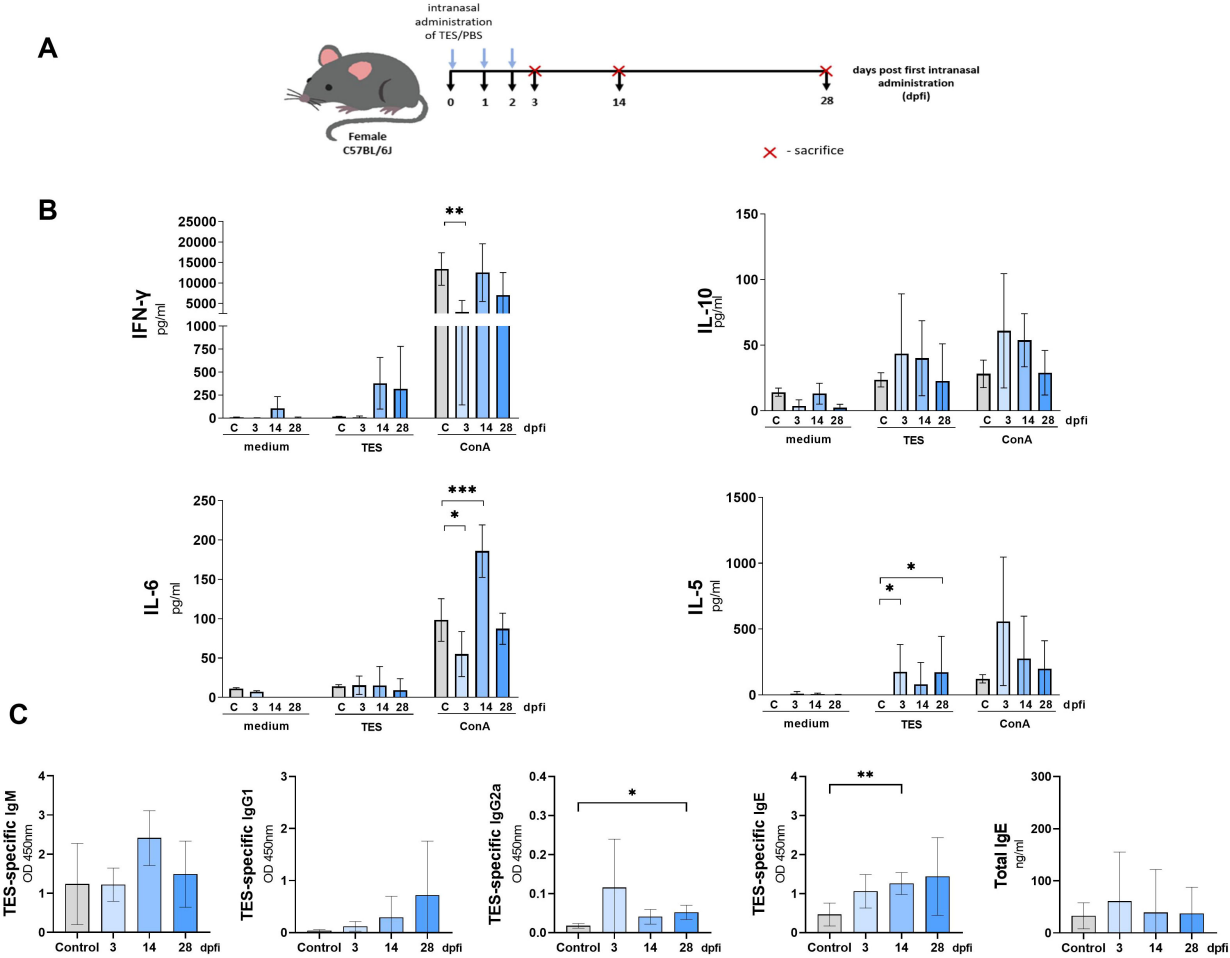
Cytokine and antibody response of mice treated intranasally with TES. **(A)** Schematic timeline representing the intranasal treatment in C57BL/6J mice. **(B)** Splenocyte cytokine response in unstimulated (medium) and TES (1ug/ml) or ConA (5 ug/ml) stimulated cultures. **(C)** TES-specific and total IgE antibody levels in mouse sera. Data is presented as mean ± SD from 5 mice in each group. Statistical differences compared to uninfected mice (0 dpi) are shown: *p < 0.05; **p < 0.01, ***p < 0.001. dpfi, days post first intranasal application.

The schematic diagram of intranasal TES administration in mice is shown in **Figure 5A**. Spontaneous cytokine release by unstimulated splenocytes did not differ between experimental groups (**Figure 5B**). However, TES restimulation significantly increased IL-5 production by splenocytes from TES-treated mice at 3 and 28 dpi. At 3 dpi, these splenocytes exhibited a weaker response to ConA stimulation, with reduced IFN-γ and IL-6 production. In contrast, at 14 dpi, IL-6 levels in response to ConA were markedly elevated (**Figure 5B**).

Intranasal TES treatment induced the production of TES-specific IgE and IgG2a (**Figure 5C**). The immunoglobulin levels were significantly higher in sera at 14 dpi and 28 dpi, respectively, compared to PBS-treated mice.

### 3.4 Intranasal TES treatment induces lung inflammation

**Figure 6:**
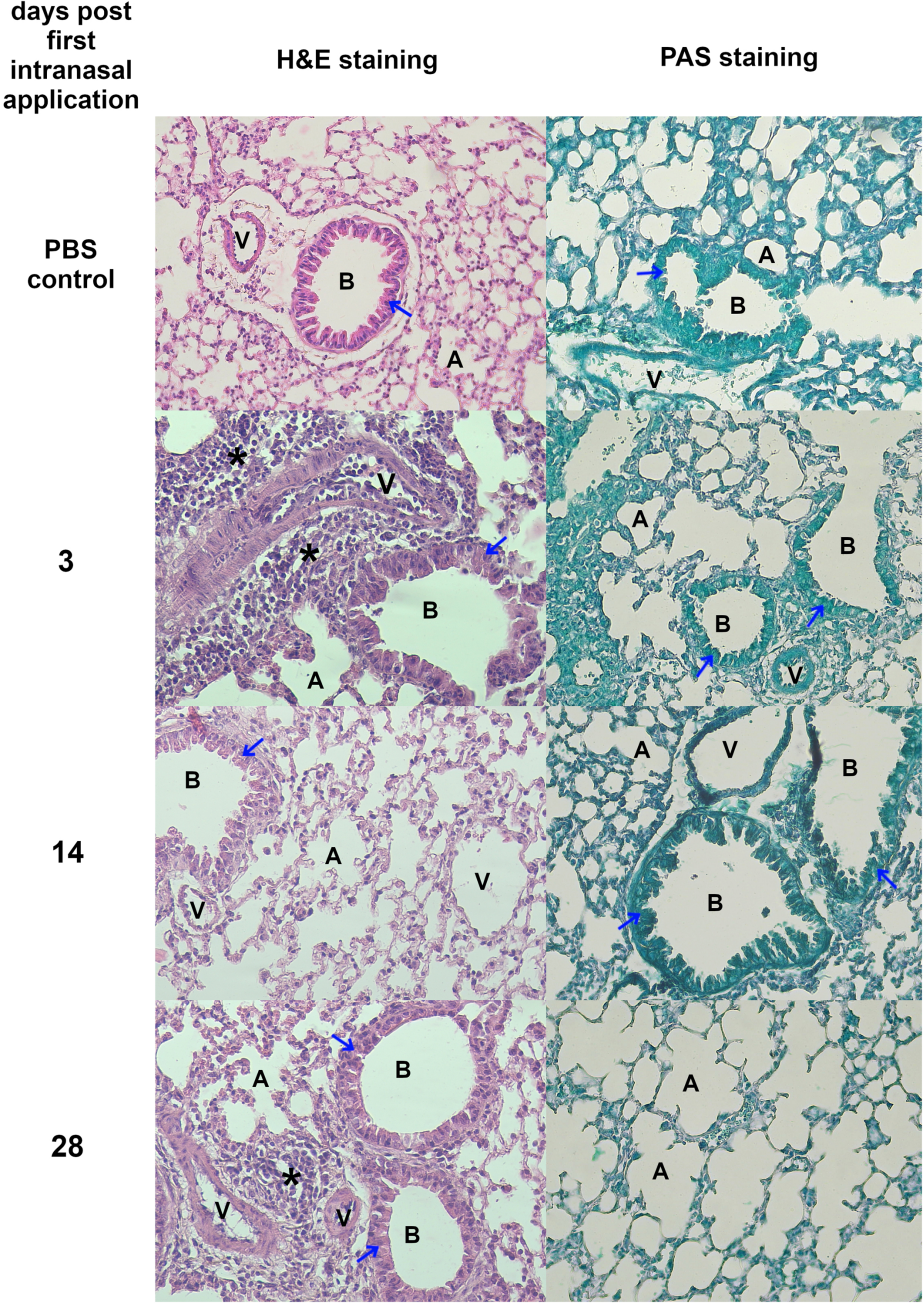
Histopathological changes in lungs isolated from mice treated intranasally with TES. Representative images of lung sections stained with H&E and PAS. All magnification ×200. Figure legend: A, alveolar lumen; B, bronchiole; V, blood vessel; * – cellular infiltrate; blue arrow – bronchial epithelial cell; red arrow – mucus; Pictures illustrating data from all experimental animals are available at https://doi.org/10.18150/83F56M.

**Figure 7:**
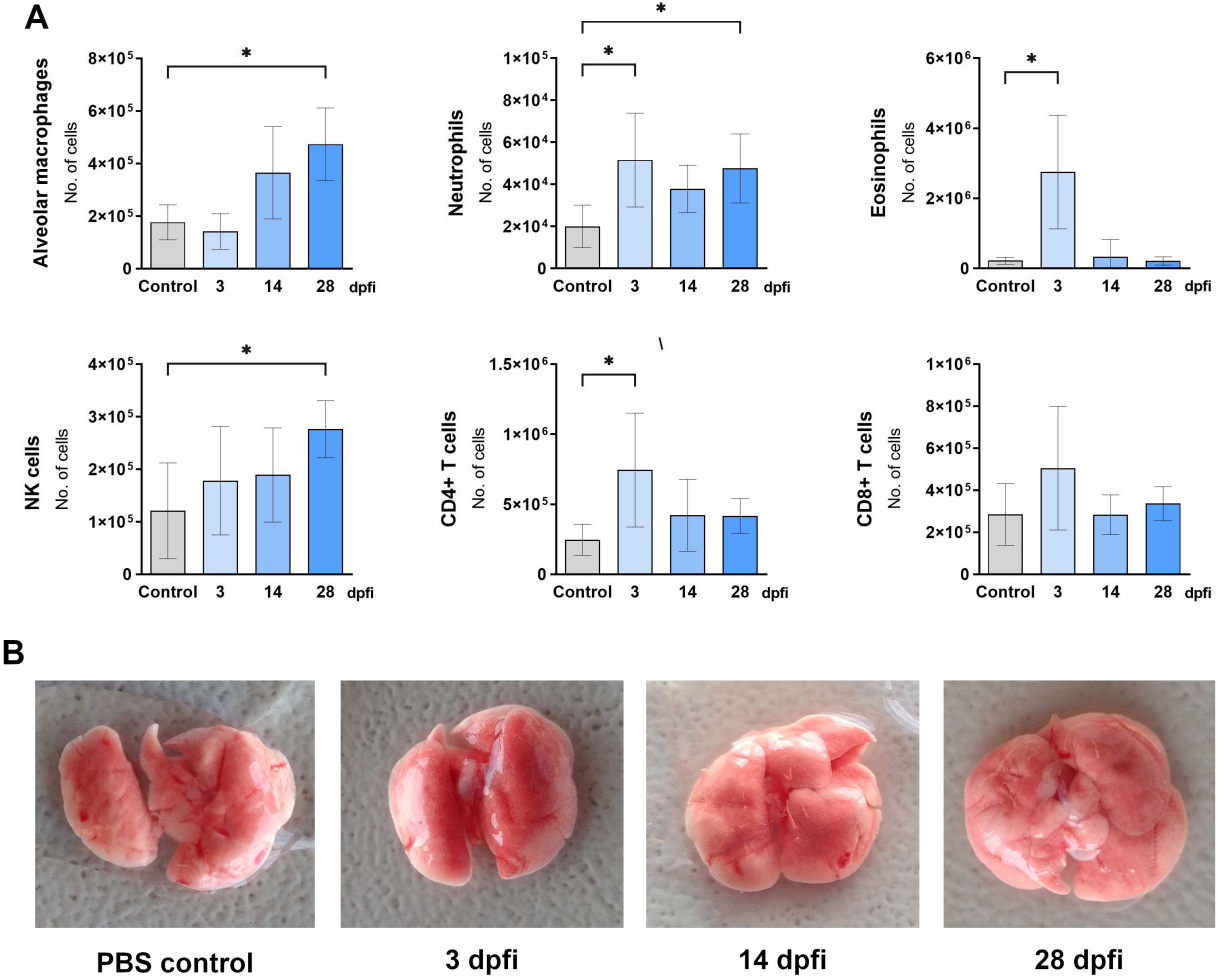
Changes in lung cell populations after intranasal TES treatment. **(A)** Absolute numbers of different lung cell populations determined using flow cytometry: neutrophils (Ly6G+CD11b+SiglecF-); alveolar macrophages (CD11c+SiglecF+); eosinophils (Ly6G+CD11b-SiglecF+); NK cells (CD49+); T helper lymphocytes (TCRαβ+CD4+); T cytotoxic lymphocytes (TCRαβ+CD8+). Data is presented as mean ± SD from 5 mice in each group. Statistical differences compared to uninfected mice (0 dpi) are shown: *p < 0.05. dpfi – days post first intranasal application. **(B)** Representative macroscopic pictures of mouse lungs at different experimental timepoints. Pictures illustrating data from all experimental animals are available at https://doi.org/10.18150/83F56M.

No visible pathological changes were observed in any group based on lung morphology evaluation. **Figures 6** and **7B** show representative pictures of lungs and histopathological analysis from each group. Pictures illustrating data from all experimental animals are available at RepOD Repository for Open Data (https://doi.org/10.18150/83F56M) However, histological differences were evident between the groups (**Figure 6**). In the control group, individual macrophages were present in the alveolar lumen, while lymphocytes and plasma cells were present around the bronchioles and in focal areas of the interalveolar septa. At 3 dpfi, there was an increase in inflammatory infiltrates, including eosinophils and mast cells surrounding blood vessels, and bronchioles. The alveolar lumen contained macrophages, neutrophils, eosinophils, and mast cells. By 14 dpfi, bronchial epithelial cells exhibited prominent eosinophilic cytoplasm and were shedding into the bronchiolar lumen, with a reduction in inflammatory cells around blood vessels and bronchioles. At 28 dpfi, only a few inflammatory cells were present around blood vessels and bronchioles and within the alveolar lumen, accompanied by the formation of a lymphoid follicle near the bronchiole. PAS staining revealed no mucus accumulation in the bronchial lumen (**Figure 6**).

During intranasal TES administration, the cellular composition of the lungs underwent the most significant change at 3 dpi, marked by an increase in eosinophils, neutrophils, and CD8+ T cells. By 14 dpi, no notable changes were detected. Interestingly, by 28 dpi, the proportions of natural killer (NK) cells, neutrophils, and alveolar macrophages had increased (**Figure 7A**).

**Figure 8:**
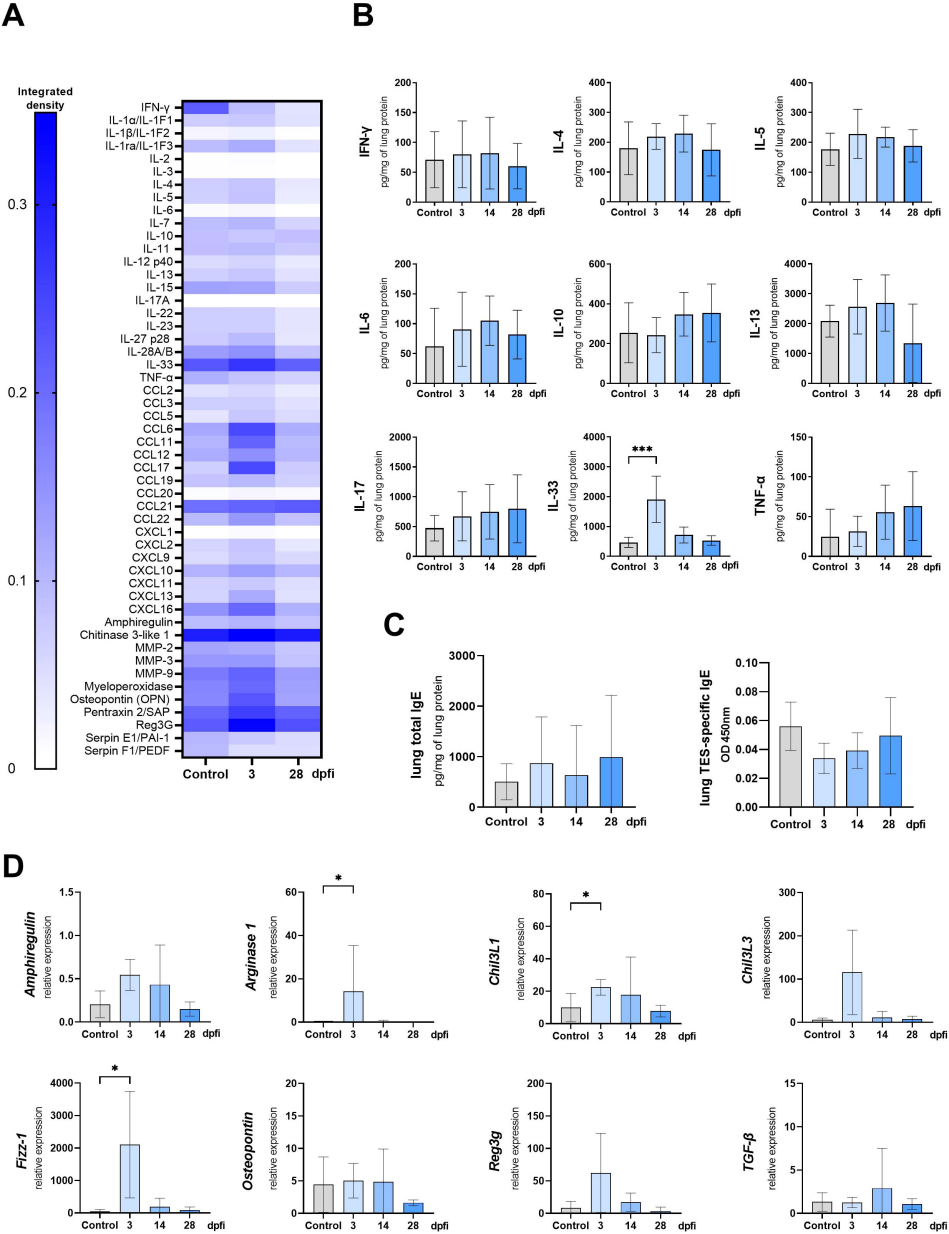
Cytokine expression and antibody changes in lungs of mice intranasally treated with TES. **(A)** Heat map representing cytokine expression in lungs determined using Proteome Profiler Mouse Cytokine XL assay **(B)** Cytokine concentrations in lung tissue lysates measured using ELISA. **(C)** Total IgE and TES specific IgE measured in lung tissue homogenates. **(D)** qPCR analysis of relative expression of selected genes involved in tissue remodeling. Results are represented as relative expression to *Hprt* housekeeping gene. Data **(B, C, D)** is presented as mean ± SD from 5 mice in each group. Statistical differences compared to control mice, which were treated with PBS instead of TES and sacrificed at 3 dpfi, are shown: *p < 0.05; ***p < 0.001. The heat map **(A)** shows the mean of integrated pixel density of two duplicate spots representing each analyte. dpfi, days post first intranasal application.

Intranasal TES treatment for three days resulted in IL-33 increase in lung tissue, as confirmed by Proteome profiler and ELISA (**Figures 8A, B**). Moreover, levels of CCL6, CCL11, CCL17, CCL22, CXCL13, and CXCL16, along with MMP-9, myeloperoxidase, osteopontin, pentraxin, and Reg3g, were also higher compared to mice treated with PBS (**Figure 8A**). By 28 dpfi, cytokine levels returned to values comparable to the control group. Intranasal application of TES also resulted in significant upregulation of *Arginase 1*, *Fizz-1*, and *Chi3L1* at 3 dpfi as shown by qPCR analysis (**Figure 8D**). The levels of TES-specific and total IgE in lungs did not change after intranasal TES application (**Figure 8C**).

## 4 Discussion

Research on the impact of *T. canis* infection on the lungs has primarily been conducted using BALB/c mice. A search in the PubMed database for *Toxocara canis, lung; C57BL* returns seven results, one of which pertains to a different *Toxocara* species, *Toxocara tanuki*, and only one study dates from the 21st century (9). In contrast, searching for *Toxocara canis, lung, BALB* yields 40 results. It is widely accepted that BALB/c mice have a stronger tendency to develop a Th2 immune response compared to C57BL/6 mice (10). However, Ly and colleagues have reported no significant differences between these two strains in shifting balance between Th1 and Th2 responses or eosinophil counts (14). Additionally, one study has reported that C57BL/6 mice exhibit lower airway inflammation compared to BALB/c mice (11). However, other investigations, such as those by Oboki et al. (2010) (12) and Entwistle et al. (2019) (13), have shown comparable or even stronger inflammatory responses in C57BL/6 mice, suggesting that strain differences may be context-dependent and influenced by specific experimental conditions (11).

Different mouse strains show varying levels of susceptibility to *T. canis* infection; some are resistant, while others maintain high numbers of larvae in somatic tissues (15). BALB/c mice appear particularly vulnerable to *T. canis* infection, as they harbor significantly more larvae than other strains. Strube et al. argue that, for this reason, BALB/c mice have been considered the most suitable model for studying toxocariasis (15). However, this raises the question of whether research should prioritize models that favor extreme cases. Given the variability in immune responses, even within different mouse strains, we chose to use C57BL/6 mice in our study. This approach aims to provide more comprehensive insights into the course of infection and to better reflect the immune response of a paratenic host.

In our study, mice were infected with *T. canis* infective eggs or intranasally treated with *T. canis* excretory-secretory products. The systemic immune response was investigated by analyzing cytokine production by spleen cells. The most prominent changes between control and infected mice were noted at 5 and 14 dpi, when IFN-γ secretion decreased while IL-5 and IL-6 levels increased, even without splenocyte restimulation. Despite its localized effect, intranasal TES stimulation could also induce a recall response, as TES-restimulated splenocytes exhibited increased IL-5 production, which persisted up to four weeks from TES application.

Interestingly, our study did not show IL-4 production by mouse splenocytes. IL-4 is considered a key cytokine in driving the Th2 response. This may be due to the particular mouse strain used in our study. Previous research comparing allergic inflammation across different mouse strains also found no increase in IL-4 levels in C57BL/6 splenocyte cultures and noted higher IL-5 secretion compared to BALB/c mice (10, 16). Studies reporting IL-4 levels in *T. canis* infected BALB/c mice are ambiguous (16–19). This raises the question of whether IL-4 production differences stem from strain-specific factors and how IL-4 contributes to the overall immune response to *Toxocara* larvae. Further investigation is needed to clarify this aspect.

Splenocyte cultures contain not only T and B lymphocytes but also innate immune cells such as monocytes, macrophages, and dendritic cells, which can secrete cytokines—including IL-10 and IL-6—even without external stimulation. This basal activity likely explains the measurable levels of these cytokines observed in control groups. An increase in IL-10 secretion was observed in TES-restimulated splenocyte cultures at 14 dpi. However, after ConA stimulation, IL-10 levels were lower than in the control group. ConA is a mitogen only for T lymphocytes (20). This finding suggests that IL-10 production following TES restimulation is driven by other spleen-derived cells. B lymphocytes are a significant source of IL-10, and this cytokine plays a crucial role in their biology, being essential for their survival, differentiation, and antibody isotype switching (21). The most intense antibody production occurs between 5 and 14 dpi, which may explain why cells from 14 dpi secrete more IL-10 upon TES restimulation.

In *T. canis* infected mice, an increase in IgG1 and IgE antibody levels, a hallmark of a Th2 response, was observed in the blood from day 14 dpi, consistent with previous studies on BALB/c mice (22–24). In TES-treated mice, the proportion of TES-specific IgE antibodies in the blood increased at 14 dpfi, although the overall IgE pool did not rise. Unexpectedly, IgM levels remained elevated at 28 days post-infection, indicating continued antigenic stimulation or delayed isotype switching. This observation is consistent with previous findings, where IgM responses peaked at day 54 before decreasing (25).

It seems that the infection had the biggest impact on IL-5 production. Its release at 5 and 14 dpi was significantly increased, even without additional restimulation. After ConA treatment, splenocytes secreted significant amounts of IL-5 as far as 28 dpi. A substantial increase in IL-5 production was also observed in mice treated intranasally with TES, but only after additional restimulation of splenocytes with larval antigens. IL-5 is classified as a typical type 2 interleukin and plays a key role in the generation, activation, and survival of eosinophils (26) in *T. canis* infection (27, 28). Interestingly, our research suggests that IL-6, a cytokine less commonly associated with parasite infections, may play a significant role in the immune response. We found that splenocytes from infected mice produced IL-6 spontaneously, potentially due to spleen and vascular damage caused by parasite migration. The peak IL-6 levels at 14 days post-infection may be linked to its role in promoting antibody production, facilitating the differentiation of activated B cells into plasma cells, and possibly influencing macrophage polarization, favoring the alternatively activated M2 macrophage phenotype (29). In bacterial pneumonia, IL-6 is considered a critical factor for integrating systemic responses to local infection, as the expression of acute-phase proteins in the liver depends on STAT3 activation by IL-6 (30). However, despite these insights, the role of IL-6 in the course of *T. canis* invasion remains unanswered.

In the context of local immune response analysis, flow cytometry revealed that eosinophils constituted the largest proportion of leukocytes in the lungs of mice from both experimental groups. Eosinophil dominance, reaching nearly 60%, persisted until 28 dpi and was observed as early as 3 dpfi in TES-treated mice. While this highlights a strong eosinophilic response during *T. canis* infection, the functional implications remain to be fully defined. Eosinophils are considered terminal effectors in allergic responses and parasite elimination. Still, they may also function as antigen-presenting cells, and are able to capture helminthic antigens in the initial phase of infections, migrate to T cell-rich regions and present antigens to trigger specific responses ( (31)). Rodolpho and colleagues showed that *T. canis* infection leads to robust activation of eosinophils and upregulation of important activation and co-stimulatory-related molecules (32). Moreover, they showed that TES antigens stimulate eosinophils isolated from non-infected mice to increase the expression of MHCII and CD69 activation markers. This proves that eosinophils may contribute to *T. canis* antigen presentation.

On the other hand, Takamoto and colleagues investigated the progression of *T. canis* infection in the lungs of IL-5 knockout mice. The absence of IL-5 reduced eosinophil infiltration in the lungs but did not impact larvae number or distribution. Interestingly, mice lacking IL-5 also exhibited less damage in lung tissue (27). Whether the massive eosinophilia triggered by *T. canis* in the paratenic host has a functional significance in host defense or rather contributes to pathology remains to be determined.

At first glance, our results from the flow cytometry analysis might indicate a decrease in macrophage numbers in the lungs following *T. canis* infection. On contrary, histopathological analysis clearly shows the presence of abundant macrophages in lung tissue sections. In our study, macrophages were identified using antibodies targeting CD11c+SiglecF+ cells. In healthy mouse lungs, alveolar macrophages (AlvMf) are characterized by high and uniform expression of CD11c, SiglecF, and CD169, whereas interstitial macrophages (IntMf) express high levels of CD11b but lack SiglecF. Under normal conditions, IntMfs constitute a small fraction of the total lung macrophage population compared to AlvMfs, but this balance can shift during lung diseases (33).

Another explanation of AlvMf number reduction could be the formation of multinucleated giant cells, commonly observed in parasitic infections (34). These cells are too large to be effectively detected by flow cytometry. In a mouse model of allergic lung inflammation, IL-4 and IL-13 have been shown to create an environment that promotes the phenotypic shift of macrophages into alternatively activated macrophages (AAM) and giant cells (35). A particularly intriguing question remains: which macrophage phenotype dominates in the lungs following *T. canis* infection? Further investigation is needed to clarify this aspect of the immune response.

Using qPCR analysis, we confirmed the upregulation of genes’ expression in the lungs that encode proteins involved in repair processes, especially *Fizz-1*, *Arginase-1*, and *Chitinase-Like Proteins* in both experimental groups. In the case of nasal TES administration, increased expression was observed only at 3 dpfi, while in the infection model, elevated expression of *Arginase-1* and *Chil3L3* persisted throughout all experiment time points. All of these proteins are synthesized by AAMs (36), and these factors are responsible for proliferation and extracellular matrix deposition (37–39). However, eosinophils—also elevated in infected lungs—can contribute to *Chi3l3* expression during Th2-type inflammation (40). In parallel, *Chil3l1* was also upregulated, which is notable given its production by eosinophils, macrophages, and epithelial cells, and its role in regulating Th2 inflammation, AAMs activation, and fibroproliferative repair. CHI3L1 also promotes eosinophil activation and migration (41), potentially amplifying the inflammatory and regenerative response. However prolonged expression of abovementioned molecules may contribute to lung fibrosis.

An intriguing role is played by Reg3g, which was elevated at 14 dpi in the lungs of infected mice. Its role is not fully understood, but significant increases in its expression have been observed in mice with subacute pneumonia induced by exposure to natural air pollution (42). Moreover, Reg3g suppresses allergic airway inflammation by inhibiting the production of epithelial cytokines – IL-33 and Thymic stromal lymphopoietin (TSLP) through STAT3 activation (43), suggesting it has a regulatory function in controlling excessive responses. Future research should focus on the duration and intensity of repair factors expression.

To the best of our knowledge, the impact of *T. canis* infection on the lungs of C57BL/6 mice, beyond histopathological analysis, has been described in only one publication by Leal-Silva et al. (44). Our study is the first to present a comprehensive cytokine proteome analysis of lung tissue during *T. canis* infection. In the available literature, cytokine concentrations are typically measured in BAL fluid (45, 46) or assessed via qPCR on lung tissue (17, 23), whereas our study employed a localized approach by analyzing lung homogenates. This method was also used by Leal-Silva et al., but their results were limited to day 3 post-infection, showing a statistically significant but modest increase in IL-5 and a pronounced increase in IL-33 (44).

In a separate study on *T. canis* infection in BALB/c mice, Leal-Silva et al. observed an increase in IL-33, IL-13, and IL-1β levels, along with a decrease in IFN-γ at 3 dpi. By 14 dpi, IL-4 and IL-5 levels had risen, and by 63 dpi, there was an additional increase in IL-10, IL-17, and IL-12 expression (22). In our study, C57BL/6J mice showed an increase in IL-33 at 3 dpi, followed by a rise in interleukin levels, but only at 28 dpi. This confirms earlier statements, that the immune response to *T. canis* differs between mouse strains.

Our study highlights how destructive *T. canis* infection can be for the lungs despite the parasite’s short presence in this organ. The highest number of *T. canis* larvae in the lungs is observed on day 3 post-infection, with only a few remaining by day 5 (13, 40). Our findings indicate that the strongest immune response in the lungs occurs at 28 dpi, whereas in TES-treated mice, inflammation is short-lived and peaks at 3 days after the first administration.

An intriguing phenomenon is the presence of mucus in the bronchial lumen at 14 dpi, despite the absence of larvae in the lungs at this time. Mucus hypersecretion is typically considered a mechanism responsible for expelling parasites from the respiratory tract (47). However, excessive mucus production is detrimental to respiratory health, as it reduces airflow and predisposes individuals to chronic infections (48). Airway obstruction due to mucus has long been recognized as a major cause of death in asthma (49). Our results suggest that mucus production is primarily triggered by mechanical damage caused by the larvae, as we did not observe increased mucus secretion following TES administration.

In our study, TES was administered for three days to mimic the duration of larval presence in the lungs. However, it is important to consider the potential role of dead larvae, which may persist in the lungs for a longer period. The impact of antigens shedded from dead parasites remains unknown. Additionally, migrating larvae produce high levels of circulating antigens. Bowman et al. reported that *T. canis*-infected mice exhibit elevated circulating antigen levels for up to eight months post-infection (50). This raises the question of whether the observed mucus production is driven by circulating TES antigens, the presence of dead larvae or the direct effects of active larval migration through the lungs. Another possibility is that larval presence in distant organs, such as muscles, the liver, or the brain, may be responsible not only for the increased mucus production but also for mobilizing the local immune response in other ways, as evidenced by the increased cytokine levels observed in the lungs at 28 dpi.

In the early phase of *T. canis* infection and intranasal TES administration, the chemokines CCL6, CCL11, CCL17, and IL-33 appear to play a significant role. Although mechanical damage caused by the parasite seems to be the primary trigger for interleukin production, TES alone induces increased IL-33 secretion. Notably, IL-33 levels following TES administration are comparable to those observed at 3 and 5 dpi.

IL-33 is produced in the lungs following damage to alveolar type II pneumocytes, the epithelial cells that contribute to the alveolar structure (51). However, it can also be secreted by immune cells, including macrophages, dendritic cells (DCs), eosinophils, B lymphocytes, monocytes, and mast cells, which likely explains its elevated levels after the early stages of infection (51). IL-33 initiates Th2 responses by stimulating the release of Th2 cytokines (primarily IL-5 and IL-13) from type 2 innate lymphoid cells (ILC2s). In our study, IL-33 seems to act directly on eosinophils, regulating their survival, activation, and adhesion (52).

However, IL-33 does not act as a chemoattractant for eosinophils; this function is fulfilled by CCL11, known as eotaxin, which is a key mediator in the development and maintenance of allergen-induced eosinophilic airway inflammation (53). CCL11 promotes eosinophil recruitment to tissues within 8–12 hours after their release from the bone marrow (54). Additionally, CCL11 contributes to tissue remodeling by enhancing fibroblast proliferation, collagen synthesis, and mast cell progenitor migration to inflamed tissue (53). CCL6 is a chemotactic factor primarily for monocytes and macrophages, but also for CD4+ T cells and eosinophils. It is known to alternatively activate macrophages, and in a murine model of allergic asthma, eosinophils have been identified as its main source (55). CCL17, secreted by lung epithelial cells, promotes the accumulation of ALVMfs in the lungs and may also regulate eosinophil migration (56). It therefore appears that CCL11 and CCL17 reach their highest levels during the early phase of infection, acting as rapid attractants for eosinophils to the site of inflammation. Subsequently, IL-33 secretion increases, supporting eosinophil survival in tissues and stimulating the release of other Th2 interleukins. In the lungs of infected mice, we observed elevated levels of above-mentioned cytokines throughout the experiment, indicating a persistent inflammatory state. Additionally, CCL6 peaks on day 28 post-infection, which is likely associated with increased activation toward the M2 phenotype, responsible for the production of repair factors such as Arginase-1 and chitinase-like proteins.

The results obtained from both the Proteome Profiler Assay and ELISA performed on lung tissue lysates, show a drop in the levels of most cytokines at 14 dpi comparing to 7 and 28 dpi. ELISA results from individual samples showed that in most cases cytokine values for three out of five mice in 14 dpi group were significantly lower, and this might explain lower mean value for the whole group. In turn, HE staining of lung tissue sections revealed a similar level of inflammatory changes in all individuals at 14 dpi. Therefore, the high variability of lung cytokine levels in mice from 14 dpi group may result from a technical error during lung lysate preparation.

The significant increase in the production of interleukins (apart from IL-33) in the lungs between infected and uninfected mice was only recorded at 28 dpi. Interestingly, in contrast to the general immune response, IL-4 was noted. This cytokine can be secreted by mast cells, Th2 lymphocytes, basophils, and eosinophils (57). Since we did not observe its release by spleen cell cultures, it seems that lymphocytes were not the primary source of IL-4, or it originated from Th2 tissue-resident memory cells, which do not migrate to the spleen (58).

In addition to the classical Type 2 response cytokines, at 28 dpi, IL-6, IL-10, IL-17, and TNF-α, are also produced. According to Proteome profiler, IL-15, IL-11, and interleukin-1 receptor antagonist (IL-1ra) are significantly elevated, which highlights the complexity of the lung response and suggests exploring not only the canonical Type 2 response. IL-17 and TNF-α synergistically induce the surface expression of IL-13Rα2 on primary lung fibroblasts, making them unresponsive to IL-13 (59). This suggests that by 28 dpi, the body is attempting to mitigate ongoing inflammation. On the other hand, IL-6 is required for excessive mucus secretion by airway epithelial cells and has been shown to inhibit Th1 differentiation independently of IL-4 (60). Although IL-6 is necessary for mucus production in the airways, likely through its effect on IL-13 production by CD4+ T cells, a direct impact of IL-6 on goblet cells in the lungs cannot be excluded (60). Given the increased production of IL-6 both systemically and locally, this cytokine appears to be an interesting target for further research related to *Toxocara* infections.

Our study has some potential limitations. Unfortunately, the nasal administration of TES has some drawbacks. It is challenging to control whether and to what extent the antigens have entered the respiratory system, and these variations may lead to significant variability in the results between individuals. Additionally, the level of IL-13 production by splenocytes was not measured, which would have provided more detailed information on Th2 response activation. This is particularly important since no IL-4 secretion by splenocytes was observed, suggesting that assessing IL-13 could have provided a clearer picture of the immune response during *T. canis* infection in C57BL/6 mice. Moreover, in terms of the systemic response, we focused on the spleen due to its role in systemic immune responses. But including blood (beyond measuring immunoglobulin levels) and lymph nodes would provide a more complete picture of immune dynamics, especially early local responses and circulating cells. We acknowledge this limitation and recommend future studies incorporate these compartments for a fuller understanding of immune activation in this model.

In conclusion, our study provides new insights into the local and systemic immune response to *T. canis* infection in C57BL/6 mice, highlighting the key role of cytokines such as IL-5, IL-6, and IL-33. Despite the absence of IL-4 production in splenocyte cultures, a Th2-type immune response was observed, characterized by eosinophil infiltration and increased levels of IgE and IgG1. However, it appears that by day 28 post-infection, the immune response is not only represented by type 2 immunity, but the Th1 component also becomes significant. The persistent mucus production and increased expression of repair-related genes suggest long-term effects on the lungs, even after the parasite has migrated from this organ. Notably, intranasal administration of TES-induced a localized immune response, partially mimicking infection, though with less intense and shorter-lived inflammation. These findings emphasize the need for further research into the immune mechanisms underlying *T. canis* infection, particularly the roles of IL-6 and IL-33, as well as the exploration of potential therapeutic targets.

## Data Availability

The original contributions presented in the study are included in the article/**Supplementary Material**. Further inquiries can be directed to the corresponding author.

## Supplementary material

Supplementary Table 1 Median or SD of the graphs.

Supplementary Figure 1 Gating strategy lymhpoid panel.

Supplementary Figure 2 Gating strategy myeloid panel.
