# Supplementary figures and images for: Characteristics of Toxocara canis induced lung inflammation in C57BL/6 mice
Source: Front Immunol. 2025 Aug 21;16:1597778. doi: 10.3389/fimmu.2025.1597778 (PMC12408637; doi:10.3389/fimmu.2025.1597778)

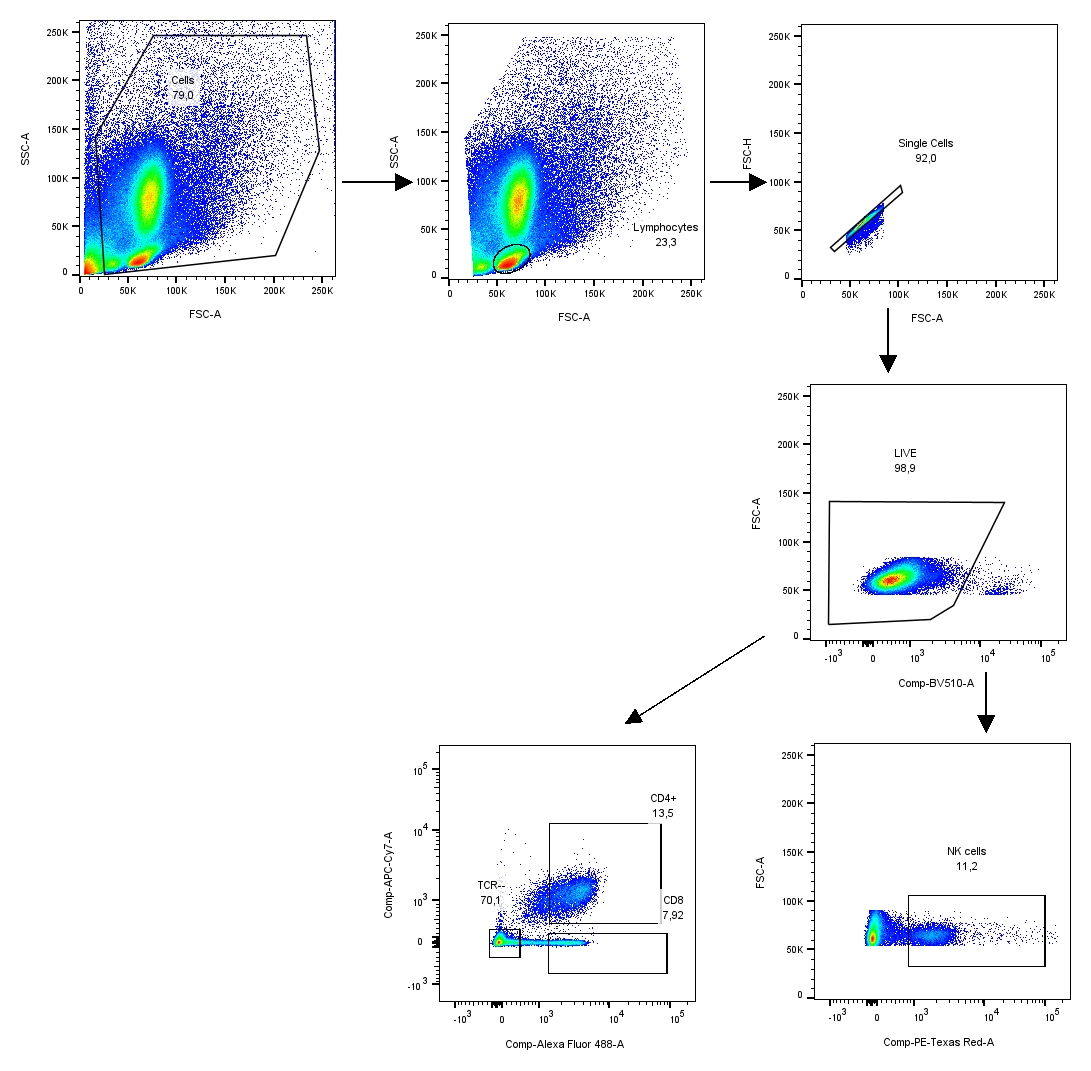

Supplement: Supplementary Figure 1 — Gating strategy lymhpoid panel. [file Image1.jpeg]

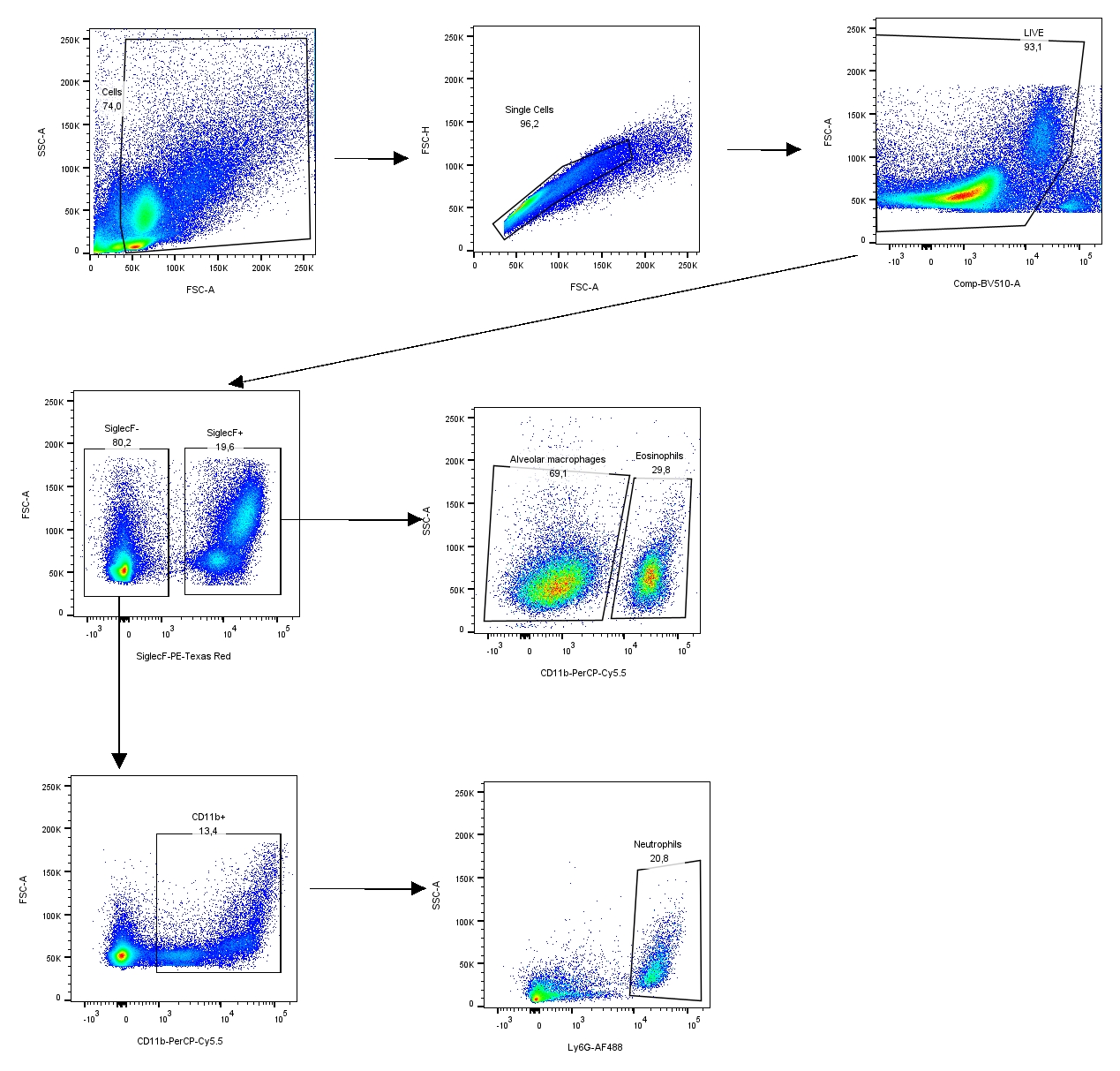

Supplement: Supplementary Figure 2 — Gating strategy myeloid panel. [file Image2.jpeg]
